# SimTune: bridging the simulator reality gap for resource management in edge-cloud computing

**DOI:** 10.1038/s41598-022-23924-0

**Published:** 2022-11-10

**Authors:** Shreshth Tuli, Giuliano Casale, Nicholas R. Jennings

**Affiliations:** 1 Imperial College London, London, UK; 2 Loughborough University, Loughborough, UK

**Keywords:** Information technology, Computer science, Software

## Abstract

Industries and services are undergoing an Internet of Things centric transformation globally, giving rise to an explosion of multi-modal data generated each second. This, with the requirement of low-latency result delivery, has led to the ubiquitous adoption of edge and cloud computing paradigms. Edge computing follows the data gravity principle, wherein the computational devices move closer to the end-users to minimize data transfer and communication times. However, large-scale computation has exacerbated the problem of efficient resource management in hybrid edge-cloud platforms. In this regard, data-driven models such as deep neural networks (DNNs) have gained popularity to give rise to the notion of edge intelligence. However, DNNs face significant problems of data saturation when fed volatile data. Data saturation is when providing more data does not translate to improvements in performance. To address this issue, prior work has leveraged coupled simulators that, akin to digital twins, generate out-of-distribution training data alleviating the data-saturation problem. However, simulators face the reality-gap problem, which is the inaccuracy in the emulation of real computational infrastructure due to the abstractions in such simulators. To combat this, we develop a framework, SimTune, that tackles this challenge by leveraging a low-fidelity surrogate model of the high-fidelity simulator to update the parameters of the latter, so to increase the simulation accuracy. This further helps co-simulated methods to generalize to edge-cloud configurations for which human encoded parameters are not known apriori. Experiments comparing SimTune against state-of-the-art data-driven resource management solutions on a real edge-cloud platform demonstrate that simulator tuning can improve quality of service metrics such as energy consumption and response time by up to 14.7% and 7.6% respectively.

## Introduction

In recent years, the technological landscape has seen a swift integration of the Internet of Things (IoT) driven infrastructures^1^. The fundamental driving factor for this transformation has been the increase in computational capacity as well the decrease in costs of computational devices^1^. As computation has become more affordable and accessible, the enormous amounts of data generated from IoT sensors and actuators have fueled the growth of paradigms such as edge and cloud computing^2^. To eschew sending all data to cloud backends that have high communication-latencies, recent solutions leverage compute resources at the *edge* of the network^3^. Having resources closer to the users facilitates the reduction of data processing times and improves the user-perceived Quality of Service (QoS)^3^. However, as the number of computing resources grows and the application workloads exhibit non-stationary fluctuations at small timescales, managing such resources becomes even more complex^4^. Static heuristic-based solutions are ineffective in such settings, and contemporary solutions rely on data-driven methods, such as Deep Learning (DL). One such DL approach, namely deep neural networks (DNNs), has become increasingly popular in managing hybrid edge-cloud platforms. DNN based methods have shown promise in effectively handling large-scale computational infrastructures, thanks to their high modeling accuracy and the ability to adapt in volatile settings if supplied enough data^4^. In this work, we leverage DNNs for effective resource management in edge-cloud computing platforms to revolutionize the computing landscape and optimize service delivery.

### Challenges

The problem of efficient resource management in edge-cloud platforms is challenging. This is prominent in the case of task scheduling, which refers to the placement of incoming tasks on available resources to optimize QoS. As the number of incoming tasks and edge-cloud devices increases, effectively scheduling tasks becomes challenging^5^. This is exacerbated by the non-stationary characteristic of most contemporary applications^6^. Even with modern neural network based solutions, most methods are unable to effectively adapt to non-stationary scenarios. In particular, as neural networks are trained on a set of pre-collected data, they tend to *learn* data patterns within the given data. Such data could be in the form of application execution traces in an edge-cloud environment, including the resource utilization characteristics, such as the CPU, Memory, Disk and Network bandwidth consumption of the running workloads on the computing devices. The trained DNNs can then predict, for instance, the utilization characteristics in a future timestep, facilitating online resource management. This also allows us to use neural networks as *surrogates* to QoS scores and as an aid to optimization^6,7^. However, DNNs tend to face the problem of *data saturation*, which is when giving more traces to the model does not improve its predictive performance^6,8^. This has been identified as a common issue in the past^8^, specifically due to the exposure bias at training time that is characteristic of the specific edge-cloud configurations used to generate the training data^6^.

### Existing methods and critique

The problem of data saturation in neural network based resource management methods has been addressed to some extent using coupled simulators, also referred to as co-simulators in the literature^6^. Other methods combine analytical methods with neural networks to simulate a physical environment^9,10^. However, such methods typically model a small set of specific aspects of a simulator, such as energy consumption or response time, using a low-fidelity DNN surrogate. Typically, such methods are not robust enough to be applicable to a generic simulator. Unlike the low-fidelity surrogates that directly predict QoS estimates for a future timestep, simulators have encoded information regarding the behavior characteristics of edge-cloud devices^11^, allowing them to perform a high-fidelity estimation of QoS scores. However, such information is typically encoded in simulators as system parameters by human experts^12,13^. These parameters include the typical communication latencies, the power consumption profiles, and task allocation overheads in edge and cloud machines^14^. In co-simulation driven methods, akin to a digital twin, an event-driven simulator is used to virtually execute multiple decisions and observe how they affect the environment and QoS scores. This enables us to explore out-of-distribution data, enabling the neural model to generalize to settings previously unseen during training and alleviate data saturation^15^. Additionally, a simulator could also facilitate injecting information regarding the system behavior within the surrogate optimization methods. Further, volatile scenarios can undergo significant shifts in the data trends, such as the resource utilization time series of running workloads. This is referred to as *high-dynamism* in edge-cloud setups^16^. Such shifts in trends can also be very frequent, referred to as *high-volatility* in edge-cloud environments^17^. Co-simulators can also facilitate obtaining additional data online without executing resource management decisions in the real infrastructure, facilitating the neural models to adapt to dynamism and volatility. However, co-simulation based methods face two critical drawbacks. First, it has been observed that the simplifications and assumptions in setting the human-encoded simulator parameter give rise to a reality gap between the estimates generated by the simulators and those from actual execution in the real environments^18^. This diminishes the credibility of using simulators to avoid data saturation as now the bias leaks into the co-simulation pipeline due to the assumptions made by human experts while settings the simulator parameters. Second, in the case of highly volatile workloads, executing several simulations may be time inefficient or computationally heavy, making it ineffective in large-scale setups with resource-limited edge devices that run such simulations.

### Contributions

In this work, we aim to address both drawbacks of co-simulation based methods highlighted above. We develop a novel framework, called *SimTune*, which aims to tune the simulator parameters to bridge the reality gap and improve simulated estimates. The key insight that we use is to leverage a DNN based surrogate model that acts as a low-fidelity twin of the simulator. This surrogate model uses the hand-encoded simulator parameters, environment and workload characteristics to generate QoS estimates. It is trained to mimic a high-fidelity simulator in terms of how close it can match the QoS estimates. Considering a trace generated from a real environment, this surrogate is then used to update the simulator parameters, both offline and online, such that the reality gap between the simulated estimates and ground-truth values is minimized. The tuned simulator parameters are then used to perform data augmentation to support data-driven schedulers. Experiments with multiple state-of-the-art schedulers on a real edge-cloud platform demonstrate that the tuned simulator can improve QoS scores compared to hand-encoded parameters and baseline tuning methods. Specifically, SimTune reduces energy consumption and response time by up to 14.7% and 7.6% respectively compared to baselines in an edge-cloud platform with DL based workloads.

### Outline

The rest of the paper presents a brief background with motivation and related work in “Background and related work” Section. “Methodology” Section presents the system model, problem formulation and the SimTune methodology. We then validate and show the efficacy of the SimTune based resource management policies in “Evaluation” Section. Finally, “Conclusions” section concludes the work and proposes future directions.

## Background and related work

Many data-driven scheduling methods have been proposed to effectively manage resources in edge-cloud computing environments. Such methods typically rely on data-driven DNNs to generate QoS estimates and run gradient-free or gradient-based optimization in the decision space to optimize objective scores.

### Scheduling methods

Most state-of-the-art scheduling methods utilize DNNs or search strategies to find optimum scheduling decisions^19^. For instance, a line of work uses evolutionary search approaches such as particle swarm optimization (PSO) using a trained DNN based QoS surrogate^20–22^. Other methods utilize genetic algorithms for QoS aware decision optimization^23,24^. Typically, such approaches run a gradient-free search scheme with non-local jumps, using cross-over and mutation-like operations to converge towards an optimum. However, gradient-free methods are known to take much longer to converge^25^ and are not as scalable^26^ as gradient-based methods. This problem is alleviated by gradient-free optimization in schedulers such as GOBI^6^ and GOSH^7^. Such methods take the scheduling decision and state of the edge-cloud system as resource utilization characteristics of workloads and Fog nodes and output a QoS estimate. Using backpropagation to input^6^, *i.e.*, fixing the neural network parameters and updating the scheduling decision based on the gradient of DNN output, these methods find the optimal scheduling decisions. However, continuous approximation of a discrete optimization problem is known to give sub-optimal decisions in some cases^27^ and thus, we consider both gradient-free and gradient-based schedulers in this work. Another category of methods is the ones that leverage reinforcement learning, popular in discrete-time control optimization settings such as distributed computing^28–30^. Such methods rely on deep neural networks that directly predict decisions instead of QoS estimates. For instance, some methods model the scheduling problem as a Markov Decision Process (MDP) and use a deep-reinforcement learning strategy, namely deep Q-Learning (DQL) to schedule workloads in a heterogeneous computing environment^28–30^. Policy gradient methods, such as^31^, train a DNN to predict the optimal scheduling decision instead of Q values directly. A recent method, Asynchronous Advantage Actor-Critic (A3C), is a policy gradient method that schedules workloads using an actor-critic pair of DNN agents^32^. These methods rely on data traces to train their models and make resource management decisions in edge-cloud environments. We consider these methods in our evaluation to test the efficacy of a tuned simulator on QoS for offline training and online fine-tuning of the neural networks used in such methods.

### Simulator tuning

The concern of the reality gap in simulators has been highlighted in the past, albeit in domains other than resource management^13,18^. The root cause of simulations being far from realism is the improper tuning of the simulation parameters for diverse scenarios that need to be simulated. For instance, in an edge simulator that simulates the energy consumption of edge devices for a given set of workloads, the power profile is often set by human experts using existing profiling data^33^. However, this profile, *i.e.*, power consumption for varying utilization of the CPU may change based on the temperature of the ambient environment, cooling solutions as well as device characteristics. Thus, having a preset profile curve may not be helpful in unseen configurations. Such simplifying assumptions give rise to the discrepancies between simulated and true metric values. Considering the growing complexity of modern simulators with millions of parameters, each with millions of possible values, running a brute-force approach is intractable. To address this, prior work has leveraged evolutionary optimization strategies such as Sim2Real^18^. Sim2Real iteratively updates the simulator parameters, performs simulations and evaluates the deviation between the simulated and true values. However, for complex simulators, running a high-fidelity simulation each time has a high computational overhead, thus, limiting the number of iterations that can be performed to update the parameters. Another method, DiffTune^13^, uses a differentiable surrogate model that can quickly generate simulated scores and can be used to update the simulation parameters. However, DiffTune can only be used to tune simulator parameters offline, where we cannot use the surrogate to perform online data augmentation and fine-tuning of the scheduling methods. DiffTune assumes a differentiable surrogate and a continuous approximation of the discrete optimization problem; this may not be ideal in most settings as many simulation parameters typically take categorical values. We solve this issue in our work by developing a deep neural network based low-fidelity simulator and using gradient based parameter update. These assumptions constrain us to utilizing a restrictive set of neural or analytical models to optimize simulation parameters. Forgoing these assumptions, and using more accurate neural models (such as with non-linear activation functions such as ReLU or sigmoid) with sub-gradients and rounding, limits the performance of such methods^34,35^. We empirically demonstrate this in "Evaluation" section.

**Figure 1:**
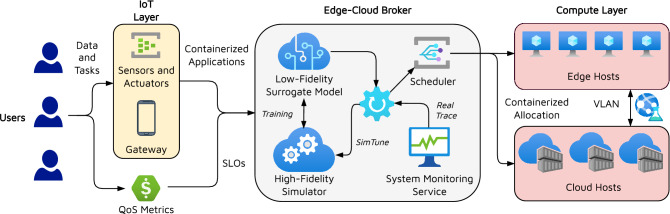
System model.

## Methodology

### System model

In this work, we assume a typical edge-cloud computing environment with multiple heterogeneous edge and cloud nodes in a broker-worker setup^6,12,24^. An overview of the system model is presented in Fig. 1. All workloads are generated in the form of data and processing tasks from the users. The data is collected through IoT sensors and passed to the computational setup via gateway devices such as smartphones and smartwatches. This is typical in smart-home or smart-hospital like environments that aim to utilize data and AI applications to process it, for instance, to run energy optimization or for patient care^4^. Each task also has expected QoS metrics such as service deadline associated. Such deadlines are also referred to as Service Level Objectives (SLOs). The tasks are realized in the form of virtualized Docker container applications to allow ease of management and secure computing^36^. The container applications and SLOs are relayed to the edge-cloud broker, which takes all resource management decisions. It monitors the edge and cloud nodes and generates traces of system characteristics such as resource utilization and QoS metrics. The broker also leverages a discrete event-driven high-fidelity simulation in tandem with a low-fidelity surrogate model to run the SimTune approach. The tuned simulator is used by the scheduler to decide optimal scheduling decisions. The decision is enacted in the physical environment in the form of container allocation or migration to respective edge or cloud nodes. Further, we assume a bounded execution timeline, which we divide into fixed-size scheduling intervals. We consider a *bag-of-task* workload model wherein a set of new tasks is created at the start of each interval and all tasks can be scheduled independently.

**Figure 2:**
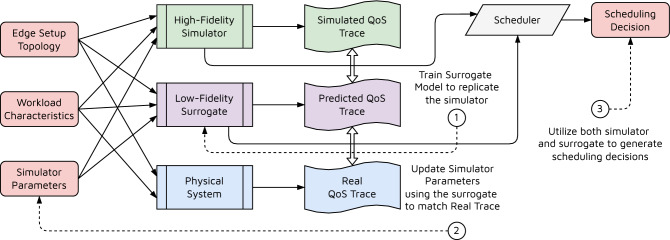
SimTune pipeline.

### Formulation

As described above, we consider a bounded timeline discretized into fixed-size intervals, each of $\Delta$ seconds. We denote the *t*-th interval by $I_t$, where $t \in \{1, \ldots , T\}$. We define the *state* of the edge-cloud environment as the collection of resource utilization metrics including CPU, RAM and Disk of the host machines. This also includes the network topology of the system as a graph with each edge consisting of parameters such as latency and network bandwidth. We denote the state at the start of interval $I_t$ by $G_t$. We also denote the time-series workload characteristics and scheduling decisions in the form of utilization of the CPU, RAM, Disk and Network bandwidth and one-hot encoding of the host allocations, up to interval $I_t$ by $W_t$. The high-fidelity simulator is denoted by a function

1 $$\begin{aligned} Q_t = f(W_t, G_t; \phi _t), \end{aligned}$$

where $\phi _t$ denotes the simulator parameters in interval $I_t$ and $Q_t$ denotes the set of QoS parameters at the end of $I_t$. Thus, the simulator acts as a high-fidelity model that simulates the scheduling decisions in $W_t$ to estimate the QoS parameters at a future timestep. We denote the *measured* QoS metrics at the end of $I_t$ by ${\bar{Q}}_t$. Considering a trace of system states, workload characteristics and QoS metrics $\mathcal {T} = \{(W_0, G_0, {\bar{Q}}_0), \ldots , (W_T, G_T, {\bar{Q}}_T)\}$, we can generate a *simulated* QoS trace by following equation (1) for each timestep *t*. This gives us a simulated trace as $\{{Q}_0, \ldots , {Q}_T\}$. Considering QoS metrics as dense vectors, we can quantify the reality-gap for a set of simulator parameters $\phi _t \forall t$ as the L2 norm

2 $$\begin{aligned} RG_t = \left\| {\bar{Q}}_t - Q_t \right\| . \end{aligned}$$

As has been observed in the past that data-driven schedulers rely on simulated estimates to optimize resource management decisions^6,7^, we need to ensure that we can bridge the reality gap to avoid bias and inaccuracies due to poorly set simulator parameters. Bridging this gap would translate to higher quality training data generated via simulators and directly translate to higher QoS scores (we demonstrate this in "Evaluation" section). Thus, our objective is to minimize the reality gap to ensure that the simulated estimates are close to the physically generated ones. This can be formulated as

3 $$\begin{aligned}&\underset{\phi _t \ge 0 \forall t}{\text {minimize.}}{} & {} \sum _{t=1}^T RG_t = \Vert {\bar{Q}}_t - Q_t \Vert \\& \text {s.t.}{} & {} {Q}_t = {f}(W_t, \phi _t, G_t; \theta ), \forall \ t. \end{aligned}$$

### SimTune

As we do not have $Q_t$ at the start of $I_t$, the above optimization problem cannot be solved offline with a collected trace of on-the-fly metric-data after each timestep. To find optimal $\phi _t$ at each timestep, we leverage a DNN based model to develop a low-fidelity surrogate model of the simulator that takes in the simulator inputs, its parameters and outputs another set of QoS estimates

4 $$\begin{aligned} {\widehat{Q}}_t = {\widehat{f}}(W_t, \phi _t, G_t; \theta ), \end{aligned}$$

where $\theta$ denotes the parameters of the neural network. Now that we have both high and low fidelity models of the physical environment, we update the simulator parameters in three steps summarized in Fig. 2. First, we update the neural network parameters ($\theta$) to minimize the loss function

5 $$\begin{aligned} L_t = \left\| Q_t - {\widehat{Q}}_t \right\| , \end{aligned}$$

for each timestep *t*. This ensures that the converged parameters, say $\theta ^*$, are such that the surrogate model *closely represents* the simulator. This allows us to utilize ${\widehat{f}}$ as a proxy of *f* and generate an estimate of $Q_t$ for a timestep *t*. Second, we fix the parameters $\theta ^*$ and update the simulator parameters $\phi _t$ to minimize the reality gap of the simulator by utilizing the reality gap of the surrogate as a proxy. Advances in optimization of neural network parameters ($\theta$) such as momentum and cosine annealing facilitate quick and scalable optimization also of $\phi _t$, which stands in contrast to typical simulators that do not allow this^6^. To do this, vanilla stochastic gradient approaches can be used to update $\phi _t$ at each timestep till convergence using

6 $$\begin{aligned} \phi _t \leftarrow \phi _t - \gamma \cdot \nabla _{\phi _t} \left\| {\bar{Q}}_t - {\widehat{f}} \left( W_t, \phi _t, G_t; \theta ^* \right) \right\| , \end{aligned}$$

where $\gamma$ is the step size. The above equation gives us an iterative rule to update the simulation parameters such that the reality gap between the trained surrogate and the real trace is minimized. However, the above assumes a continuous relaxation of categorical simulation parameters such as the number of cores in a host machine (a natural number) or whether hardware acceleration or hyper-threading is supported by a machine (binary value). To circumvent this, we use Gradient-Directed Monte-Carlo (GDMC) optimization^37^. To do this, we perform discrete perturbations to $\phi _t$ and build a tree from the current value. Each node in the tree is represented as an ordered pair $(v, \phi _t, n)$ where $\phi _t$ is the parameter value set, $v^i$ is a value estimate and *n* is the frequency of visits to that node. Each node has multiple child nodes $\{(\phi _t^i, n^i)\}_i$ where we select a node in each Monte-Carlo selection stage such that the Upper-Confidence-Bound

$$\begin{aligned} v^i - \nabla _{\phi _t} \left\| {\bar{Q}}_t - {\widehat{f}} \left( W_t, \phi _t, G_t; \theta ^* \right) \right\| - \sqrt{\frac{c \ln {n}}{n_i}}\end{aligned}$$

is minimized, where *c* is an exploration parameter. The $\nabla _{\phi _t} \Vert {\bar{Q}}_t - {\widehat{f}}( W_t, \phi _t, G_t; \theta ^* ) \Vert$ term aims to select nodes in the direction of the gradient and the $\sqrt{\frac{c \ln {n}}{n_i}}$ term ensure other perturbations are also explored. As we select child nodes, we calculate $v^i = \Vert {\bar{Q}}_t - {\widehat{f}}( W_t, \phi _t^i, G_t; \theta ^* ) \Vert$. After each such computation for a leaf node of the tree, we backpropagate values such that *v* becomes the frequency weighted average of $v^i$’s of its child nodes. As we perform multiple roll-outs and visit frequencies increase, the third term diminishes in value and we perform higher exploitation than exploration. Finally, we choose the simulation parameter with the highest $v^i$. Performing the above iteratively to update $\phi _t$ we obtain $\phi ^*_t$ such that the reality gap of the surrogate is minimized. Finally, we use the $\phi ^*_t$ parameters to generate data using the simulator to train the data-driven scheduler. We can also leverage the low-fidelity surrogate to perform on-the-fly $\phi _t$ optimization to generate $\phi ^*_t$ at each interval.

This pipeline offers three key advantages compared to prior work. First, as a neural network offers quick and scalable inference, we can also leverage it for online data generation and tuning of the scheduler. Second, optimization methods based on backpropagation to input have shown promise in the past and have a significant advantage of being able to optimize inputs quickly to minimize simulation tuning time. Third, with each incoming data point, we can fine-tune $\theta$ and then simulator parameters $\phi _t$ to dynamically adapt the simulation parameters as the workload and system characteristics change with time.

**Figure 3:**
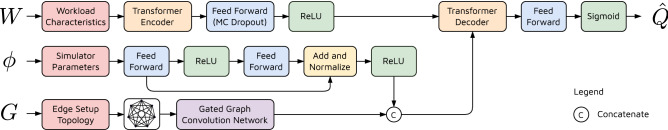
Low-Fidelity surrogate of the simulator in SimTune in the form of a neural network. The inputs include workload characteristics as time-series values, simulator parameters and edge topology as a fully-connected graph. The output is a vector of QoS estimates.

### Low-fidelity surrogate model

As described in "Introduction" section, we realize the low-fidelity model using a deep neural network. An overview of the neural network architecture is presented in Fig. 3. In the rest of the discussion we drop the subscript that identifies timestep *t* without loss in generality. To infer the temporal trends in the workload characteristics, we leverage a Transformer model for their improved learning efficiency^38^. A Transformer is a multi-head attention based neural model that has been shown to be more scalable than classical recurrent modelling approaches^38^. Thus,

7 $$\begin{aligned} \begin{aligned} W^1&= \textrm{TransformerEncoder}(W),\\ W^2&= \textrm{ReLU}(\textrm{FeedForward}(W^1)). \end{aligned} \end{aligned}$$

However, in this feed-forward network, we use Monte-Carlo Dropout (MCD) for Bayesian inference at test time^39^. Unlike conventional dropout, MCD enables dropout at inference time as well. This allows us to run inference multiple times and obtain a stochastic output, specifically to model the volatile nature of workload characteristics. To infer the simulator parameters, we use a feed-forward network,

8 $$\begin{aligned} \begin{aligned} \phi ^1&= \textrm{ReLU}(\textrm{FeedForward}(\phi )),\\ \phi ^2&= \textrm{ReLU}(\textrm{LayerNorm}(\textrm{FeedForward}(\phi ^1) + \phi ^1)). \end{aligned} \end{aligned}$$

where the $\textrm{LayerNorm}$ operation normalizes the output for stable training. The skip-connection between the output of the first feed-forward network and the second facilitates faster propagation of gradients and improved accuracy^40^. We also infer over the system state, *i.e.*, the edge topology graph using a graph neural network^41^. We first form a fully connected graph with all hosts represented as graph nodes. The characteristics of the host $h^j$ are denoted by $e^j$. We then pass the graph through a gated-graph convolution network to capture the inter-host dependencies rising from the new task allocation. Here, the features for host $h^j$ are aggregated over all other hosts in the graph over *r* convolutions, resulting in an embedding $e^{j}_{r}$ for each host node in the graph. Specifically, the gating stage is realized as a Gated Recurrent Unit (GRU) resulting in *graph-to-graph* updates^41^ as:

9 $$\begin{aligned} \begin{aligned} e^j_{0}&= \textrm{tanh} \left( W\ e^{j} + b \right) ,\\ x_q^j&= \sum _{j} W^q e^{j}_{q-1} ,\\ e_q^{j}&= \textrm{GRU} \left( e_{q-1}^j, x_{q}^{j} \right) , \end{aligned} \end{aligned}$$

where the second equation performs the convolutions of the features of immediate neighbors in the graph. However, for large-scale graphs, to ensure that we capture the inter task and host correlations, we perform the above convolution step *r* times. Here, a GRU is a recurrent neural network that decides the weightage of the output of the previous convolution iteration with respect to the latest iteration. This allows the model to efficiently scale with the size of input graph without significantly losing performance. The stacked representation for all hosts is represented as $G^1$. The three encodings are then concatenated and send to the Transformer decoder to generate a vector of QoS metrics as

10 $$\begin{aligned} \begin{aligned} E^1&= \textrm{TransfomerDecoder} \left( W^2, \phi ^2, G^1 \right) ,\\ {\widehat{Q}}&= \textrm{Sigmoid} \left( \textrm{FeedForward} \left( E^1 \right) \right) . \end{aligned} \end{aligned}$$

The sigmoid activation function makes the output in the range (0, 1), giving us normalized QoS scores. Overall, the objective of the neural network is to infer the simulated QoS metrics using the workload characteristics, simulator parameters and system information.

### Offline scheduler training

To train the surrogate model, we use a random scheduler and random perturbations to the simulator parameters to generate a dataset trace $\{(G_t, \phi _t, W_t, Q_t)\}_t$. This enables us to cover a large input state-space. Using such a trace, we can train a surrogate model ${\widehat{f}}(W_t, \phi _t, G_t; \theta )$ by minimizing the loss function

11 $$\begin{aligned} L = \sum _t L_t = \sum _t \left\| Q_t - {\widehat{f}}(W_t, \phi ^*_t, G_t; \theta ) \right\| , \end{aligned}$$

to give converged network parameters $\theta ^*$. Using this, and a trace from a physical system $\{(G_t, W_t, \phi _t, {\bar{Q}}_t)\}_t$, we tune simulator parameters to get $\phi ^*_t$. The model is trained using normalized QoS metrics from the simulator and real-systems. Now that we have a high-fidelity scheduler *f* and a low-fidelity surrogate ${\widehat{f}}$, we can utilize them to train a scheduler *g* that generates scheduling decisions for an input state of the system. We denote the scheduling decision for interval $I_t$ by $D_t = g(G_t, W_t)$. As such schedulers are typically data-driven, we leverage traces using random schedulers generated by the *tuned* simulator $f(\cdot ; \phi ^*_t)$. Using such a simulator, we generate a trace of system states and simulated QoS estimates as $\{(G_t, W_t, Q_t)\}_t$ to train *g*.
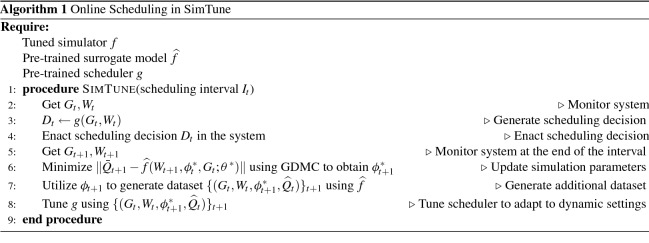


### Online scheduling

We now describe how the SimTune framework aids in informed decision making. An overview is presented in Algorithm 1. Having a trained scheduler *g*, we generate scheduling decisions at the start of each interval $I_t$ as $D_t = g(G_t, W_t)$ (line 3). To account for dynamism in the system, at the end of each interval $I_t$, we form another datapoint by estimating QoS ${\widehat{Q}}_{t+1}$ for given state $(G_{t+1}, W_{t+1})$ using $\phi ^*_t$ and updating $\phi ^*_t$ to $\phi ^*_{t+1}$ by minimizing the surrogate reality gap $\Vert {\bar{Q}}_{t+1} - {\widehat{f}}(W_{t+1}, \phi ^*_{t}, G_t; \theta ^*) \Vert$ (lines 5–6). This allows us to dynamically tune simulator parameters to minimize the reality gap online. Using the new parameter set $\phi _{t+1}$, we generate additional dataset $\{(G_t, W_t, \phi ^*_{t+1}, {\widehat{Q}}_t)\}_{t+1}$ to fine-tune the scheduler model *g* (lines 7–8). Note that we utilize the QoS estimates of the surrogate ${\widehat{f}}$ to ensure that multi-step simulation traces can be generated quickly, minimizing overall decision time of the framework. This additionally allows us to make decisions informed on the new simulator parameters and consequently the updated system trends.

## Evaluation

### Testbed

We consider a hybrid edge-cloud computing setup with 16 Raspberry Pi 4B nodes, 8 with 4GB RAM and another 8 with 8GB RAM each. This allows the setup to have heterogeneous nodes with different memory capacities. Our cloud environment consists of 34 virtual machines provisioned from Microsoft Azure cloud platform. We use diverse VM types in our cloud infrastructure, *i.e.*, B2s with a dual-core CPU and 4GB RAM, B4ms with a quad-core CPU and 16GB RAM and B8ms with an octa-core CPU and 32GB RAM. We consider a geographically distributed cloud environment. Our environment consists of 20 VMs in the UK-South Azure datacenter and 14 in the East-US datacenter. The UK-South cluster consist of 10 B2s and 10 B4ms nodes, while our East-US cluster consists of 7 B4ms and 7 B8ms nodes. Our resource management policies are run on a cloud broker node in the UK-South location and is a D16asv4 node with a 16 core CPU and 64 GB RAM. The execution costs are taken from Azure pricing calculator^42^. The power consumption values of increments of 10% CPU utilization of Azure VM types are taken from the COSCO simulator^6,43^, which includes the power consumption characteristics of B2s, B4ms and B8ms Azure VMs derived from the Standard Performance Evaluation Corporation (SPEC) benchmark repository^44^. We ignore the power consumption characteristics of the cooling infrastructure as in prior work^6,14^. As these power characteristics may be on out-of-distribution workloads and we use the fraction of incoming workload requests as a proxy of CPU utilization, the absolute values of the reported energy consumption in this paper are rough estimates.

### Workloads

In order to evaluate the performance of SimTune, we use the AIoTBench benchmarks^45^. This is a widely used AI-based computing benchmark suite that consists of various real-world computer vision application instances^46^. The seven specific application types correspond to the CNN neural networks for image classification. These include three typical heavy-weight networks: ResNet18, ResNet34, ResNext32x4d, as well as four light-weight networks: SqueezeNet1-0, GoogleNet, MobileNetV2, MnasNet. These neural models are from various industry applications, showing that this benchmark captures real-world workloads. In terms of the data to process, we use 50 images from the COCO dataset^47^. We use the interval duration in our experiments, *i.e.*, $\Delta$ as 5 minutes as per prior work^6,30,48^. To evaluate the proposed method in a controlled environment, we abstract out the users and IoT layers described in "Methodology" section and use a discrete probability distribution to realize tasks as container instances. Thus, at the start of each scheduling interval, we create new tasks from a Poisson distribution with $\lambda = 1.2$, sampled uniformly from the seven applications^6^. The distribution is a natural choice for a bag-of-tasks workload model, common in edge environments^49,50^. Each task has an associated SLO deadline generated from prior work^6^. When the response time exceeds its deadline, we call that a violation of the SLO. Our tasks are executed using Docker containers. We run all experiments for 100 scheduling intervals, with each interval being 300 seconds long, giving a total experiment time of 8 hours and 20 minutes. We average over five runs and use diverse workload types to ensure statistical significance in our experiments.

**Figure 4:**
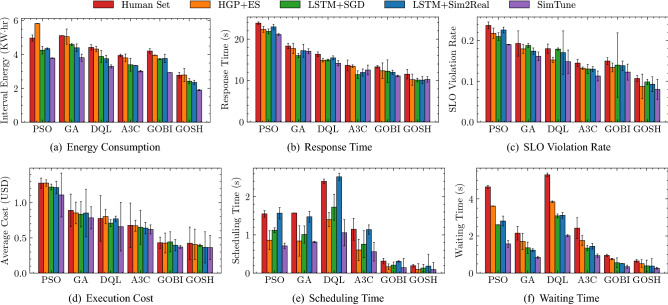
Comparison of QoS parameters (averaged over intervals) of SimTune against baseline methods.

### Model training and assumptions

For training, we randomly split the dataset into 80% training and 20% testing data. We use a learning rate of $10^{-4}$ with a weight decay of $10^{-5}$ in the Adam optimizer for optimizing the loss function. The learning rate parameter was set as per grid-search to minimize the reality gap loss mentioned in equation (11). We use the early stopping criterion for convergence.

### Baselines

We compare SimTune against four baselines as described below. Each of these methods is for tuning the simulator parameters, which we compare against SimTune across six state-of-the-art schedulers: PSO, GA, DQL, A3C, GOBI and GOSH (see "Background and related work" section for more details). These have been selected to cover diverse classes of scheduling strategies. PSO and GA are search based schedulers, whereas DQL and A3C utilize reinforcement learning. GOBI and GOSH use neural network based surrogate models to run gradient optimization and find near-optimal scheduling decisions. All schedulers were aimed to minimize the normalized energy consumption and average response time of completed tasks^6^.

- *Human Set* uses the preset simulator parameters to generate the offline and online training data required by the scheduling methods.
- *HGP+ES* uses a heteroskedastic Gaussian Process (HGP)^51^ as a low-fidelity surrogate of the simulator and evolutionary search (ES) strategy^18^ to tune the simulator parameters.
- *LSTM+SGD* uses a Long-Short-Term-Memory (LSTM) neural network with differentiable activation functions motivated from DiffTune^13^. It also uses stochastic-gradient-descent (SGD) to perform gradient optimization of simulator parameters.
- *LSTM+Sim2Real* also uses an LSTM neural network to act as a low-fidelity surrogate of the simulator with a mutation-crossover based evolutionary search strategy to optimize simulator parameters as done in Sim2Real^18^. Akin to Sim2Real and DiffTune methods, *HGP+ES*, *LSTM+SGD* and *LSTM+Sim2Real* utilize the high-fidelity simulator to generate the on-the-fly data for online scheduler training. We also do not dynamically tune the simulator parameters in these methods^13,18^.

### Evaluation metrics

To test the efficacy and performance improvement of the SimTune approach, we compare the end-term unnormalized QoS metrics. We compare the energy consumption of the edge-cloud testbed described above in terms of KW$\cdot$hr averaged over the 100 intervals for which we run an experiment. We also measure the average response time of the completed tasks in the system. Both energy consumption and response time metrics help us to distinguish the efficacy of the schedulers for each tuning (or preset) simulator. We also compare the average SLO violation rates of completed tasks. Further, we compare the execution cost (in US Dollars) of cloud machines in terms of the pay-per-use cost as well as the energy consumption cost of edge devices. We amortize the overall cost with the number of completed containers. Finally, we also compare the overheads of the decision making models by observing the average waiting time of tasks to get allocated to a host and the average scheduling time of each approach.

**Table 1:** Ablation analysis.

Approach	Scheduler					
	PSO	GA	DQL	A3C	GOBI	GOSH
**Interval Energy (KW**$\cdot$**hr)**						
SimTune w/o HiFi	4.772 ± 0.283	4.572 ± 0.360	3.772 ± 0.133	3.428 ± 0.258	4.113 ± 0.186	2.411 ± 0.236
SimTune w/o LoFi	4.262 ± 0.330	4.252 ± 0.267	3.750 ± 0.198	3.334 ± 0.146	3.505 ± 0.045	2.443 ± 0.245
SimTune	**3.788 ± 0.020**	**3.815 ± 0.212**	**3.295 ± 0.094**	**3.016 ± 0.049**	**2.931 ± 0.007**	**1.900 ± 0.033**
**Response time (s)**						
SimTune w/o HiFi	22.800 ± 0.407	18.649 ± 0.374	16.371 ± 0.635	12.669 ± 1.830	13.945 ± 0.722	11.434 ± 0.166
SimTune w/o LoFi	22.471 ± 0.842	17.294 ± 0.833	15.044 ± 0.421	12.818 ± 0.316	11.780 ± 0.607	10.489 ± 0.563
SimTune	**21.112 ± 0.201**	**17.067 ± 0.606**	**14.179 ± 0.610**	**12.560 ± 1.175**	**11.119 ± 0.196**	**10.287 ± 0.663**
**SLO violation rate**						
SimTune w/o HiFi	0.215 ± 0.003	0.177 ± 0.000	0.182 ± 0.015	0.123 ± 0.007	0.141 ± 0.005	0.100 ± 0.011
SimTune w/o LoFi	0.205 ± 0.015	0.166 ± 0.005	0.166 ± 0.005	0.126 ± 0.027	0.129 ± 0.013	0.090 ± 0.005
SimTune	**0.190 ± 0.001**	**0.161 ± 0.011**	**0.148 ± 0.029**	**0.113 ± 0.012**	**0.123 ± 0.020**	**0.080 ± 0.025**
**Average execution cost (USD)**						
SimTune w/o HiFi	1.360 ± 0.066	0.833 ± 0.345	0.737 ± 0.032	0.625 ± 0.041	0.425 ± 0.146	0.426 ± 0.109
SimTune w/o LoFi	1.154 ± 0.076	0.862 ± 0.253	0.739 ± 0.333	0.697 ± 0.129	0.403 ± 0.195	0.399 ± 0.167
SimTune	**1.108 ± 0.311**	**0.784 ± 0.157**	**0.658 ± 0.344**	**0.620 ± 0.064**	**0.370 ± 0.023**	**0.362 ± 0.171**
**Scheduling time (s)**						
SimTune w/o HiFi	0.763 ± 0.207	0.871 ± 0.329	1.235 ± 0.066	0.563 ± 0.217	0.155 ± 0.101	**0.090 ± 0.003**
SimTune w/o LoFi	1.449 ± 0.201	1.737 ± 0.262	2.469 ± 0.194	1.256 ± 0.326	0.315 ± 0.074	0.205 ± 0.158
SimTune	**0.712 ± 0.072**	**0.814 ± 0.021**	**1.056 ± 0.342**	**0.559 ± 0.243**	**0.145 ± 0.236**	0.095 ± 0.185
**Waiting time (s)**						
SimTune w/o HiFi	1.957 ± 0.445	0.928 ± 0.160	1**.878 ± 0.225**	0.985 ± 0.086	0.378 ± 0.099	0.254 ± 0.450
SimTune w/o LoFi	3.130 ± 0.244	1.277 ± 0.028	3.189 ± 0.146	1.521 ± 0.103	0.568 ± 0.019	0.399 ± 0.214
SimTune	**1.576 ± 0.179**	**0.851 ± 0.064**	2.021 ± 0.058	**0.944 ± 0.091**	**0.348 ± 0.086**	**0.268 ± 0.051**

Best values are shown in bold.

**Figure 5:**
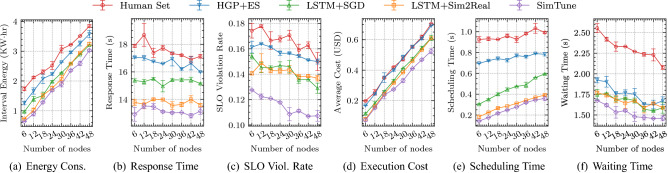
Scalability Analysis of SimTune and baselines with different number of nodes in the edge-cloud setup.

### Comparison with baselines

Figure 4 compares the QoS metrics of SimTune with the baseline methods for each of the state-of-the-art schedulers. SimTune outperforms the baselines across most metrics, such as reducing energy consumption and response time by up to 14.7% and 7.6%, respectively. Specifically, in terms of energy, *LSTM+Sim2Real* has the lowest consumption of 3.714 KW$\cdot$hr across all baselines, averaged across all schedulers. SimTune reduces this to 3.124 KW$\cdot$hr, *viz*, 14.7% lower than the best baseline. SimTune is able to provide improved energy efficiency compared to the *human-set* values thanks to the ability to tune the power profile characteristics of the edge and cloud hosts. Without simulator tuning, the data-driven schedulers are trained using the SPEC power profiles, which may not be ideal representations of the power consumption characteristics given the different workload nature of AIoTBench applications. Moreover, in terms of average response time, the *LSTM+SGD* baseline gives 15.45 s amortized across all schedulers. SimTune gives a lower response time of 14.28 s, 7.6% lower than *LSTM+SGD*. The improvements in energy consumption and response time are primarily due to the lower reality gap measured in terms of the loss function in Eq. (11). SimTune’s converged loss value is 0.0345, whereas the lowest loss among the baselines is of the *LSTM+Sim2Real* approach with 0.1922. This is due to the ability of SimTune to capture both temporal trends using a Transformer encoder as well as spatial correlations utilizing a gated-graph convolution network. The low loss value ensures that the data generated by the simulator and the surrogate are closer to the ground-truth values, alleviating the exposure bias problem and furnishing more *realistic* data to the schedulers. This directly translates to better QoS scores. Lower response times if SimTune also leads to significant improvements in terms of SLO violation rates. The lowest average violation rate among all baselines is achieved by *HGP+ES* of 0.153, whereas SimTune gives an average SLO violation rate of 0.135, 11.8% lower than the best score. Similarly, we see that SimTune gives the lowest average execution cost of 0.650 USD, which is 7.86% lower than the most cost-efficient baseline, *i.e.*, *LSTM+Sim2Real* with an average cost of 0.706 USD. Compared to stochastic gradient descent, the GDMC based parameter updates enables SimTune to update categorical parameters as well. *LSTM+SGD* uses continuous relaxation of the parameter updates instead, which has been shown to perform poorly compared to GDMC^37^. This is enabled by the Monte-Carlo Dropout for Bayseian inference from the low-fidelity neural network model. Finally, we also see lower scheduling times for the SimTune approach, thanks to the low-fidelity surrogate being used to generate on-the-fly data for dynamic scheduler training (line 7 in Algorithm 1). This also translates to lower average waiting times for the incoming tasks.

### Ablation analysis

To test the importance of the hybrid approach of SimTune that utilizes both a high-fidelity simulator and low-fidelity surrogate, we modify the approach as follows. First, we consider a model without the high-fidelity simulator to generate offline training data and utilize the surrogate itself. We refer to this approach by *SimTune w/o HiFi*. Second, we replace the low-fidelity surrogate model with the high-fidelity simulator to generate data for online training of the scheduler in SimTune. We call this approach as *SimTune w/o LoFi*. Table 1 presents the results of SimTune and the ablation models. Without the high-fidelity simulator for offline training (*SimTune w/o HiFi*), we observe a drop in the QoS metrics; for instance, SLO Violation rates increase by 5.4%. This is due to the lack of unexplored simulator configurations by the surrogate leading to poor offline training of the schedulers. Further, without the surrogate (*SimTune w/o LoFi*), the online data generation is more time-consuming, which gives rise to higher scheduling and wait times and poorer QoS scores. For instance, SLO violation rates increase by 13% when we do not use low-fidelity model. This demonstrates the effectiveness of the hybrid high and low fidelity approach of SimTune.

**Figure 6:**
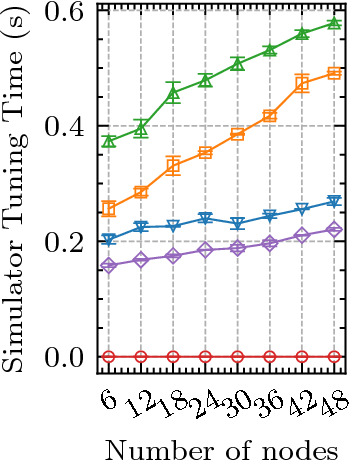
Tuning time of SimTune and baseline models with different number of nodes in the edge-cloud setup.

### Scalability analysis

To test the performance of the SimTune approach at different scales we generate QoS scores for diverse sizes of the edge-cloud testbed. We perform a controlled experiment with 1:2 ratio of edge and cloud nodes where we use equal number of 4GB and 8GB RAM Raspberry Pi 4B nodes in our edge environment and B2s cloud VM type in UK-South. We vary the number of edge devices from 2 to 16, with number of cloud nodes being 4 to 32. This gives the total number of nodes from 6 to 48. We keep the $\lambda$ parameter proportionate to the size of the setup. For the 6 node setup $\lambda = 0.16$ and for the 48 node setup $\lambda = 1.28$. The scores with different sizes of the network, averaged over all scheduling methods, are shown in Fig. 5. As the number of nodes increase, so does the energy consumption and execution cost. However, SimTune gives the lowest energy consumption and operational costs across all models. Response time and SLO violation rates do not show significant deviation for the baseline models. Due to the high chance of contention in case of limited number of devices, we see that response time and consequently SLO violation rates are typically higher for 9 or less nodes. Even in such cases SimTune gives better scores compared to baselines. This is primarily due to the ability of the gated graph convolution network to effectively scale performance with the size of the input graph^52^. We also compare the tuning time of the simulator parameters for the different number of nodes in the setup in Fig. 6. When the simulator parameters are static and *Human Set*, there is no tuning overhead. However, this limits its performance in non-stationary settings where dynamic parameter updates may be required. Within the parameter update based methods, SimTune has the lowest tuning time thanks to its Transformer based design that allows us to furnish all tuning data together in lieu of the auto-regressive inference style in recurrent models such as *LSTM+SGD* and *LSTM+Sim2Real*.

## Conclusions

This paper proposes SimTune, a framework to bridge the reality gap between the simulated and ground-truth QoS traces. SimTune leverages a low-fidelity neural network based surrogate model to tune the parameters of a high-fidelity simulator. The SimTune approach trains the neural network surrogate to mimic the simulator and updates the simulator parameters using the surrogate reality gap as a proxy and updating parameters using a gradient-based Monte-Carlo search strategy. The updated parameters are then used to generate offline data using the simulator and online data using the surrogate to train a data-driven scheduler. Experiments with real-life AI-based benchmark applications on a heterogeneous edge-cloud testbed show that SimTune gives at least 14.7% lower energy consumption, 7.6% lower response times and 11.8% lower SLO violation rates compared to state-of-the-art baselines. This demonstrates the importance of simulator tuning for optimal QoS in the domain of edge intelligence. Future work would investigate the application of the SimTune approach to also include additional resource management decisions such as resource provisioning and autoscaling^53^. We also aim to explore the application of SimTune in the domain of fault-tolerant computing.

## Data Availability

All data and code used for the current study are available from the corresponding author on reasonable request.

## Code availability

The code and relevant training scripts are made publicly available on GitHub under BSD-3 licence at https://github.com/imperial-qore/SimTune with a snapshot of the paper version available at https://doi.org/10.5281/zenodo.7273916.
